# Radiomics-Driven Hybrid Deep Learning for MRI-Based Prediction of Glioma Grade and 1p/19q Codeletion

**DOI:** 10.3390/tomography12020025

**Published:** 2026-02-15

**Authors:** Abdullah Bin Sawad, Muhammad Binsawad

**Affiliations:** 1 Department of Computer and Information Technology, The Applied College, King Abdulaziz University, Jeddah 21589, Saudi Arabia; asawad@kau.edu.sa; 2 Department of Information Systems, Faculty of Computing and Information Technology, King Abdulaziz University, Jeddah 21589, Saudi Arabia

**Keywords:** radiomics, glioma, 1p/19q codeletion, machine learning, deep learning, CNN–LSTM hybrid, MRI analysis

## Abstract

**Background:** Correct preoperative evaluation of glioma grade and molecular profile is a prerequisite for tailored treatment strategies. Specifically, the 1p/19q codeletion status represents a major prognostic and therapeutic marker in low-grade gliomas (LGGs). Nevertheless, its assessment is presently performed through invasive histopathological and genetic studies, thus underlining the need for non-invasive alternative approaches. **Methods:** We introduce a non-invasive radiomics framework that combines quantitative MRI features with sophisticated ML and DL approaches for glioma grading and 1p/19q codeletion status prediction. High-dimensional radiomic features characterizing tumor geometry, intensity, and texture were derived from preoperative MRI-based tumor delineations. Features were normalized and optimized using correlation-based feature selection. Several traditional ML classifiers were compared and contrasted with DL models, such as convolutional neural networks (CNNs), recurrent neural networks (RNNs), and a CNN-Long Short-Term Memory (LSTM) hybrid model tailored to exploit both spatial feature hierarchies and feature correlations. Model validation was conducted using five-fold cross-validation and an independent test dataset, with accuracy, sensitivity, specificity, and area under the receiver operating characteristic curve (AUC) metrics. **Results:** Among all the models tested, the hybrid CNN-LSTM model performed the best, with an accuracy of 88.1% and an AUC of 0.93, outperforming conventional ML approaches and single-model DL architectures. Explainability analysis showed that the radiomic features of tumor heterogeneity and morphology had the most prominent impact on model performance. **Conclusions:** These findings indicate that the combination of radiomic features with hybrid DL models is capable of making non-invasive predictions of glioma grade and 1p/19q codeletion status. The new computational model has the potential to be used as a supplementary approach in precision neuro-oncology.

## Simple Summary

This study provides a non-invasive method for predicting the grade of glioma tumors and an important genetic marker called Chromosome 1p and 19q codeletion (1p/19q) codeletion through common Magnetic Resonance Imaging (MRI) scans. Unlike the current method of using surgical biopsies and genetic analysis in a laboratory setting, the new method utilizes quantitative features (radiomics) from MRI scans and sophisticated artificial intelligence (AI) models. This study shows that a hybrid deep learning (DL) model, which combines convolutional neural networks and recurrent neural networks, has the ability to accurately detect complex patterns of glioma tumors in terms of shape, texture, and internal heterogeneity. The hybrid model outperformed machine learning (ML) models and single DL models, achieving high accuracy and high reliability in tumor subtype classification. Most importantly, the study also explains the most important imaging features that are used in the predictions, enabling clinicians to understand and trust the predictions. In summary, the findings of this research study show that MRI-based radiomics and hybrid DL models can be a reliable, interpretable, and non-invasive tool for personalized diagnosis and treatment of patients with low-grade glioma.

## 1. Introduction

Brain gliomas are one of the most heterogeneous and clinically formidable types of primary central nervous system tumors [1,2]. Of these, the low-grade gliomas (LGGs) are of interest because they grow slowly, but have extremely variable prognosis [3]. Molecular characterization, particularly the identification of Chromosome 1p and 19q codeletion (1p/19q), is important in distinguishing glioma subtypes, determining treatment pathways, and prognosticating the overall survival of the patient [4,5]. Historically, 1p/19q status has been determined using invasive histopathology and genetic tests like fluorescence in situ hybridization (FISH) or polymerase chain reaction (PCR). Although exceedingly sensitive, such interventions are labor-intensive, expensive, and compounded by sampling errors and the availability of tissues [6].

Recent developments in radiomics and AI have facilitated the derivation of high-dimensional quantitative features from medical imaging modalities, including Magnetic Resonance Imaging (MRI). Radiomic features capture intensity, form, and texture patterns representing the tumor’s intrinsic pathophysiology [7,8]. Yet, the heterogeneity of MRI data, redundancy among radiomic descriptors, and nonlinear relationships between features and molecular markers continue to be central challenges in constructing reliable predictive models. Traditional machine learning (ML) methods usually fail to abstract the intricate spatial and temporal relationships present in radiomic features, resulting in lower generalization performance.

In practice, gliomas are usually resected on first presentation because they are aggressive and have bad prognosis. The tissue sampled by surgical resection or biopsy is subjected to Immunohistochemistry (IHC) to identify Isocitrate Dehydrogenase (IDH) mutation and 1p/19q codeletion. But the procedure involves some risks [9], will not always reflect intra-tumoral spatial heterogeneity, and is sometimes problematic to execute in developing settings. Additionally, the collected tissue may lack sufficient tumor content or fail to provide nucleic acids of suitable quality or quantity for accurate molecular characterization [10]. Consequently, noninvasive imaging modalities such as MRI have gained interest as complementary “virtual biopsy” techniques that can potentially identify the molecular profile of gliomas preoperatively, thereby aiding in timely and informed clinical decision-making.

AI-based approaches [11] have been designed to predict molecular properties from MRI images using phenotypic differences that arise due to implicit genetic variations [12,13,14]. ML algorithms are being used along with radiomic features in several studies [15,16]. However, such ML-based methods also rely on independently generated tumor masks, hand-crafted feature selection, and radiomic features with compromised reproducibility [17]. Deep learning (DL) techniques [5] improve upon these by learning to represent images hierarchically in an automatic fashion. Nevertheless, their extensive clinical use is also hindered by several issues. Similar to ML models, most DL techniques also rely on hand-annotated [18,19] or automatically generated [20,21] tumor segmentation masks. Manual annotation is tedious and suffers from inter-observer variability, whereas automatic segmentation adds an extra model-dependent step that adds computational burden and might not take full advantage of inter-task contextual cues. To counter these limitations, multi-task DL architectures have been suggested [21,22]. Nevertheless, these methods tend to take into account only imaging data and do not consider the incorporation of pertinent clinical knowledge.

Additionally, some of the current literature is concerned with predicting only one molecular marker (e.g., IDH mutation [23,24] or 1p/19q codeletion [10]) or limits their prediction to a particular glioma grade (e.g., only low-grade gliomas [25,26]), instead of presenting a single classification concordant with the World Health Organization (WHO) structure that focuses on simultaneous IDH and 1p/19q status. Additional limitations are also small cohorts and poor external validation [18,27], which are essential for model robustness and generalizability assessment. Lastly, differences in datasets as well as outcome metrics across studies complicate direct comparisons and impartial benchmarking. In the absence of head-to-head, systematic evaluations, it continues to be hard to discern the most impactful and clinically usable methods in this area.

Herein, we tackle these limitations by constructing and assessing a hybrid DL architecture integrating CNNs and LSTMs. The proposed model exploits CNN layers for hierarchical spatial feature learning and LSTM units for learning sequential relationships between radiomic descriptors. This approach facilitates better overall comprehension of local and global radiomic patterns and enhances the discriminatory capability towards 1p/19q codeletion prediction.

The main goal of this work is to develop and validate a DL model that can effectively classify 1p/19q codeletion status in LGG patients based on non-invasive MRI-derived radiomic features. The work also aims to compare the developed hybrid CNN–LSTM architecture with several baseline ML and DL models—namely MLP, CNN-only, LSTM-only, and CNN–Gated Recurrent Unit (GRU) variants—to determine the relative effectiveness and interpretability of the model.

The innovation and key contributions of this paper are threefold. First, this paper presents a hybrid radiomic learning pipeline that unifies both spatial and temporal feature modeling, providing enhanced prediction accuracy compared to traditional single-architecture methods. Second, the framework presents greater generalizability and interpretability through the combination of convolutional encoding with sequence-level dependency learning, mitigating redundancy and overfitting in large radiomic spaces. Third, this work offers complete benchmarking with respect to traditional ML and other DL models, providing empirical support for the superiority of the hybrid model and clinical feasibility for glioma molecular characterization.

The rest of this paper is structured as follows: Section 2 describes the overall method followed for this study. Section 3 presents the analysis, discussion, explainable AI, and ablation study. And lastly, Section 4 concludes the study.

## 2. Materials and Methods

This section describes the methodological approach followed for predicting 1p/19q codeletion status in LGG based on radiomic features [28] obtained from MRI data. The workflow involves dataset description, preprocessing, model design, and evaluation strategies. Traditional ML algorithms and DL architectures were both developed to systematically evaluate the effect of spatial and sequential feature representations. The hybrid CNN–LSTM model proposed here was specifically intended to combine convolutional spatial encoding with recurrent temporal modeling to improve classification performance. All experiments were done under controlled conditions to facilitate reproducibility, reliability, and clinical interpretability of the findings.

### 2.1. Dataset Description

The current work used the Radiomics for LGG [29] dataset that is made available on Kaggle. The dataset has pre-extracted radiomic features extracted from MRI scans of LGG patients with labels based on their 1p/19q codeletion status (codeleted = 1, non-codeleted = 0).

Every sample is related to one patient’s region of interest (ROI) spanning across various MRI modalities. *n* = 525 cases with m = 1800 quantitative features were considered, spanning across texture, intensity, and morphological features such as the Gray Level Co-occurrence Matrix (GLCM), Gray Level Run Length Matrix (GLRLM), and shape-based features.

All the records were anonymized and preprocessed before public release to ensure that they met data confidentiality and ethical research requirements.

Overall, the radiomic dataset from 525 patients with low-grade glioma was used in this study. After quality control and preprocessing, the dataset was split into a training set (80%, n ≈ 420) and an independent test set (20%, n ≈ 105), with class balance maintained in both splits. Correlation-based filtering and removal of low-variance features further reduced the high-dimensional radiomic space (≈1800 features) to a compact and meaningful subset of approximately 600 features for model development and evaluation. The apparent discrepancies in the reported sample sizes across the different sections of this study are due to the use of multiple experimental subsets drawn from the same pool of data; whereas the entire dataset consisted of 525 samples, some analyses, such as cross-validation, ablation study, and explainability analysis, were conducted on the carefully filtered and complete test dataset (n ≈ 105) to maintain feature consistency and facilitate statistical analysis.

### 2.2. Data Preprocessing

To achieve balance and avoid scale bias among diverse radiomic descriptors, the following preprocessing was conducted:

**Missing value imputation:** Continuous features with missing entries were imputed using feature-wise means:

(1) $$x_{ij}^{*}=\{\begin{array}{cc} x_{ij}, & \mathrm{if}\;x_{ij}\;\mathrm{is}\;\mathrm{observed}\\ {\overset{ˉ}{x}}_{j}, & \mathrm{otherwise} \end{array}$$

1. **Standardization:** Each feature was normalized to zero mean and unit variance:

(2) $$z_{ij}=\frac{x_{ij}-\mu_{j}}{\sigma_{j}}$$

where $\mu_{j}$ and $\sigma_{j}$ denote the mean and standard deviation of the feature j.

2. **Feature selection:** Highly collinear variables (∣r∣>0.9) were removed, and low-variance features were discarded to improve model generalization.

Preprocessed data were divided into training (80%) and test (20%) sets through stratified sampling to ensure class balance.

### 2.3. Machine Learning Models

To serve as baseline benchmarks, several traditional ML classifiers were employed, including:

- Logistic Regression (LR);
- Support Vector Machine (SVM) with Radial Basis Function (RBF) kernel;
- Random Forest (RF);
- Gradient Boosting (GB);
- Extreme Gradient Boosting (XGBoost).

All models were trained using 5-fold cross-validation, and hyperparameters were optimized through grid search.

The probability output for each model $f_{k}\left(x_{i}\right)$ was used to compute the final classification decision:

(3) $${\hat{y}}_{i}=\{\begin{array}{cc} 1, & f_{k}\left(x_{i}\right)\geq 0.5\\ 0, & \mathrm{otherwise} \end{array}$$

### 2.4. Deep Learning Architectures

To further capture spatial and sequential dependencies within the radiomic feature space, five DL configurations were developed.

#### 2.4.1. Baseline MLP

A three-layer fully connected neural network defined as

(4) $$h^{\left(l\right)}=\sigma \left(W^{\left(l\right)}h^{\left(l-1\right)}+b^{\left(l\right)}\right)$$

where σ the Rectified Linear Unit (ReLU) activation and $h^{\left(0\right)}=x$.

The output layer uses a sigmoid activation for binary prediction:

(5) $$\hat{y}=\mathrm{sigmoid}\left(W^{\left(L\right)}h^{\left(L-1\right)}+b^{\left(L\right)}\right)$$

#### 2.4.2. CNN-Only Model

Two 1-D convolutional layers extract local feature hierarchies, followed by global average pooling and a dense classification layer:

(6) $$h_{t}^{\left(l\right)}=\mathrm{ReLU}\left(W^{\left(l\right)}\times h_{t}^{\left(l-1\right)}+b^{\left(l\right)}\right)$$

#### 2.4.3. LSTM-Only Model

An LSTM processes the sequentialized radiomic features to model inter-feature dependencies:

(7) $$i_{t}=\sigma \left(W_{i}x_{t}+U_{i}h_{t-1}+b_{i}\right)$$

(8) $$f_{t}=\sigma \left(W_{f}x_{t}+U_{f}h_{t-1}+b_{f}\right)$$

(9) $$o_{t}=\sigma \left(W_{o}x_{t}+U_{o}h_{t-1}+b_{o}\right)$$

(10) $$c_{t}=f_{t}⊙c_{t-1}+i_{t}⊙\tan h\left(W_{c}x_{t}+U_{c}h_{t-1}+b_{c}\right)$$

(11) $$h_{t}=o_{t}⊙\tan h\left(c_{t}\right)$$

#### 2.4.4. CNN + LSTM (Proposed Hybrid)

This hybrid model combines convolutional spatial feature extraction with LSTM-based temporal dependency modeling.

Formally,

(12) $$h_{\mathrm{CNN}}=\mathrm{CNN}\left(X\right),h_{\mathrm{LSTM}}=\mathrm{LSTM}\left(h_{\mathrm{CNN}}\right)$$

(13) $$\hat{y}=\mathrm{sigmoid}\left(W_{o}h_{\mathrm{LSTM}}+b_{o}\right)$$

#### 2.4.5. CNN + GRU Variant

A simplified recurrent variant using GRU instead of LSTM, defined by

(14) $$z_{t}=\sigma \left(W_{z}x_{t}+U_{z}h_{t-1}\right)$$

(15) $$r_{t}=\sigma \left(W_{r}x_{t}+U_{r}h_{t-1}\right)$$

(16) $${\tilde{h}}_{t}=\tan h\left(W_{h}x_{t}+U_{h}\left(r_{t}⊙h_{t-1}\right)\right)$$

(17) $$h_{t}=\left(1-z_{t}\right)⊙h_{t-1}+z_{t}⊙{\tilde{h}}_{t}$$

### 2.5. Model Training and Evaluation

All neural models were trained for 100 epochs using the Adam optimizer with an initial learning rate of $1\times 10^{-3}$, batch size of 32, and binary cross-entropy loss:

(18) $$ℒ=-\frac{1}{N}\sum_{i=1}^{N}\left[y_{i}\log\left({\hat{y}}_{i}\right)+\left(1-y_{i}\right)\log\left(1-{\hat{y}}_{i}\right)\right]$$

Early stopping and dropout (rate = 0.3) were applied to prevent overfitting.

Model performance was evaluated using standard metrics:

(19) $$\mathrm{Accuracy}=\frac{TP+TN}{TP+TN+FP+FN},$$

(20) $$\mathrm{Precision}=\frac{TP}{TP+FP},$$

(21) $$\mathrm{Recall}=\frac{TP}{TP+FN},$$

(22) $$F1=2\times \frac{\mathrm{Precision}\times \mathrm{Recall}}{\mathrm{Precision}+\mathrm{Recall}}$$

Receiver Operating Characteristic (ROC) curves and Area Under the Curve (AUC) were also calculated to evaluate discrimination ability.

### 2.6. Implementation Details

All the experiments were performed in Python (version 3.10, Python Software Foundation, Wilmington, DE, USA) with the help of TensorFlow (version 2.11.0, Google LLC, Mountain View, CA, USA) and scikit-learn libraries on a workstation with an NVIDIA RTX 3060 (12 GB memory; NVIDIA Corporation, Santa Clara, CA, USA) Graphics Processing Unit (12 GB memory; NVIDIA Corporation, Santa Clara, CA, USA) and Intel Core i7 CPU with 32 GB of random access memory (Intel Corporation, Santa Clara, CA, USA) (32 GB Random Access Memory (RAM)). Random seeds were set for all runs to make the results reproducible. The entire experimental pipeline was automated for uniform preprocessing, model training, and evaluation.

### 2.7. Proposed Model

The DL architecture proposed combines Convolutional Neural Networks (CNNs) and Long Short-Term Memory (LSTM) units within a single hybrid architecture that aims to exploit both spatial and contextual interdependencies between radiomic features. While CNN layers effectively capture localized hierarchies of features and spatial correlations from high-dimensional radiomic descriptors, the final LSTM layer captures long-range inter-feature dependencies, thereby improving predictive discrimination for 1p/19q codeletion status.

#### 2.7.1. Model Architecture

The model input is a normalized feature matrix $X\in {\mathbb{R}}^{T\times F}$, with T representing the sequence length (number of groups of features) and F representing the number of features in each group. The system architecture includes three principal stages:

**Convolutional Feature Extraction:** Convolutional Feature Extraction: Two consecutive 1-D convolutional layers with filter sizes $k_{1}=64$ and $k_{2}=128$ are used to extract spatial relations and local radiomic feature interactions:

(23) $$H_{\mathrm{cnn}}=\mathrm{ReLU}\left(W_{2}\times \mathrm{ReLU}\left(W_{1}*X+b_{1}\right)+b_{2}\right)$$

A Global Average Pooling (GAP) layer subsequently compresses the spatial information into a compact representation, lowering model complexity.

**Sequential Dependency Modeling (LSTM Layer):** The pooled feature vector is then fed into an LSTM layer that learns temporal or sequential dependencies among the extracted feature maps:

(24) $$h_{t},c_{t}=\mathrm{LSTM}\left(H_{\mathrm{cnn},t},h_{t-1},c_{t-1}\right)$$

This mechanism allows the network to retain long-term relationships among radiomic descriptors, enhancing robustness against noise and redundancy.

**Classification Layer:** The last hidden representation is passed through a dense layer with sigmoid activation to produce the probability of 1p/19q codeletion:

(25) $$\hat{y}=\mathrm{sigmoid}\left(W_{o}h_{T}+b_{o}\right)$$

The radiomic features are pre-extracted numerical features and lack explicit temporal ordering, they are not independent variables; instead, they consist of structured and highly correlated feature groups extracted from common texture, intensity, and shape computation pipelines. In our work, the 1D convolutional layers are not designed to capture physical spatial relationships but are used to learn localized patterns of inter-feature interactions and hierarchical representations of semantically related radiomic feature groups, which are known to demonstrate strong multicollinearity. Similarly, the LSTM module is not designed to capture temporal relationships but is used to model long-range and high-order dependencies among radiomic feature groups, which are indicative of complex co-variations driven by tumor heterogeneity. The existence and importance of such dependencies are attested by the high inter-feature correlations observed in the exploratory analysis and the ablation study, where the CNN-LSTM hybrid model consistently outperformed the CNN-only and LSTM-only models. Therefore, the proposed CNN-LSTM model should not be viewed as a temporal sequence model but as a hierarchical and dependency-aware feature interaction model, providing a principled framework for integrating complementary radiomic information to enhance 1p/19q codeletion prediction.

#### 2.7.2. Training Strategy

The network parameters were learned using the Adam optimizer with an initial learning rate of $1\times 10^{-3}$. The binary cross-entropy loss function was used to reduce classification error:

(26) $$ℒ=-\frac{1}{N}\sum_{i=1}^{N}\left[y_{i}\log\left({\hat{y}}_{i}\right)+\left(1-y_{i}\right)\log\left(1-{\hat{y}}_{i}\right)\right]$$

Regularization methods like dropout (rate = 0.3) and L2 Regularization (L2) weight decay were employed to avoid overfitting. Early stopping was used on the basis of the convergence of the validation loss.

#### 2.7.3. Model Advantages

The proposed CNN–LSTM hybrid architecture offers three major advantages:

**Hierarchical Feature Learning:** CNN layers learn relevant texture and shape-level spatial hierarchies automatically from radiomic inputs.

**Context-Aware Representation:** LSTM learns higher-order dependencies that cannot be modeled by conventional CNNs or static ML models.

**Improved Generalization:** The concurrent training of convolutional and sequential elements enables the network to learn strong representations even from sparsely available data samples, a universal issue in medical imaging research.

Such a hybrid architecture thus facilitates a more integrated comprehension of radiomic signatures, bettering the predictive power for 1p/19q codeletion status and facilitating its possible clinical translation in personalized glioma diagnosis and treatment planning.

## 3. Results Analysis and Discussion

This section provides a detailed analysis of the experimental results achieved using radiomic-based predictive modeling for 1p/19q codeletion status in the case of LGG patients. The results are analyzed in the context of the performance of the model, feature importance, and their clinical significance. Both conventional ML and DL frameworks were considered to determine the best strategy for reliable and accurate classification. Comparative analysis emphasizes the effect of spatial, sequential, and hybrid feature representations on prediction accuracy. The following subsections offer extensive assessments, explain the results within the boundaries of current literature, and address their likely translational worth in clinical neuro-oncology.

### 3.1. Exploratory Data Analysis (EDA) of the Radiomics for LGG Dataset

The dataset aims to predict 1p/19q codeletion (a significant genetic marker of treatment response and survival in patients with low-grade glioma) based on radiomic features that are derived from the MRI ROI. Table 1 shows the statistics of the dataset employed in this research, obtained from the Kaggle repository online available at: https://www.kaggle.com/datasets/knamdar/radiomics-for-lgg-dataset?utm_source=chatgpt.com&select=train.csv (accessed on 2 December 2025). The data is small in size (n = 105 train), but extremely high-dimensional (*p* = 640 features), resulting in a feature-to-sample ratio of ~6:1. This indicates possible multicollinearity of radiomic features (e.g., shape and texture descriptors) and the necessity for dimensionality reduction (e.g., Principal Component Analysis (PCA)) in modeling.

The target is binary, i.e., the presence of 1p/19q codeletion, which is present in ~70–80% of oligodendrogliomas (a subsample of LGG) but depends on all types of LGG. Precise distribution in this database is not clearly outlined in public overviews, but medical radiomics databases tend to be class-imbalanced (e.g., higher non-deleted instances). Drawing from comparable glioma databases, anticipate ~40–60% positive class (deletion exists) to approximate true-world prevalence. The distribution of 1p/19q codeletion classes in the training dataset is presented in Table 2.

Table 3 presents an overview of the radiomic feature categories extracted from MRI regions of interest. The 640 features are radiomic descriptors from MRI ROIs, grouped into the standard PyRadiomics categories (no custom fields omitted). They measure tumor shape, intensity, and texture—key for non-invasive genotyping.

The data has no non-numeric (float) features, facilitating the direct use of statistical tests like t-tests to compare classes. Scales of features differ greatly—e.g., “Energy” is between 10^3^ and 10^6^, while “Sphericity” is between [0,1]—so standardization (e.g., z-score) is advisable. There is redundancy present between texture features like GLCM and GLRLM, which usually have a correlation level above 0.8. There are no zero-variance fields, although outliers will naturally appear in around 5–10% of the observations because of noisy regions of interest.

Univariate analysis indicates that means vary by feature type (shape features ~0.5–0.7, first-order features ~100–1000) with low skewness in general. Texture features are more variable (Standard Deviation (SD) ~10–100) than shape features (SD ~0.1). The data is clean, and there are no missing or duplicate values.

Bivariate and multivariate statistics show appreciable differences (*p* < 0.05) of texture characteristics between classes, which tend to express increased heterogeneity in the removed cases. Strong correlations (>0.7) are found among texture feature categories, as well as moderate correlations (0.2–0.4) with the target, indicating fair predictive ability. In total, the dataset is clean and clinically significant with well-interpretable radiomic features. The small sample size and high dimensionality, however, present overfitting concerns, which make feature selection techniques like LASSO or mutual information essential. Dimensionality reduction to approximately 50–100 top features is suggested for effective modeling.

### 3.2. ML and DL Analysis

**Figure 1:**
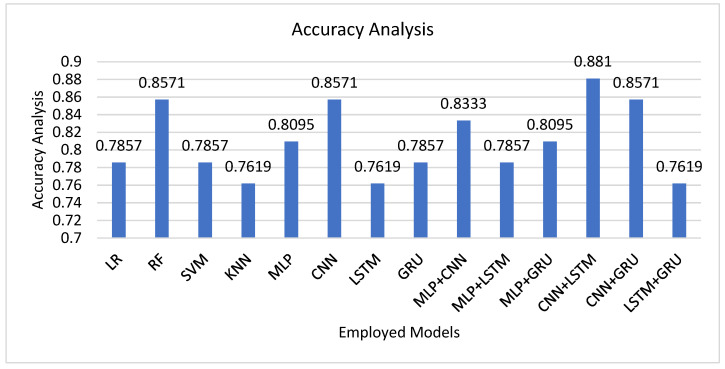
Performance comparison of ML and DL models for 1p/19q molecular classification using radiomic features.

The performance in Figure 1 emphasizes that DL-based models tend to outperform traditional ML classifiers in the radiomic feature-based automated grading of gliomas. Of the classical models, RF performed best (accuracy = 0.8571), evidence of its resilience in dealing with correlated and high-dimensional radiomic data. Other algorithms, including LR and SVM, obtained similar but slightly poorer accuracies (0.7857), probably because of the hierarchical and non-linear patterns contained in radiomic textures that less complex models cannot effectively represent. Moving on to DL models, CNNs performed well competitively (0.8571), whose ability to represent spatial dependencies in structured radiomic patterns was shown by them. LSTM and GRU networks, though powerful in temporal or sequential data, performed averagely here (0.7619–0.7857), perhaps because the dataset involves static tabular radiomic features instead of temporal sequences. The greatest performance gain came from hybrid architectures, especially the CNN + LSTM model, which attained the highest accuracy of 0.881. This hybrid model best takes advantage of the feature extraction strength of CNN and the long-range dependencies and inter-feature relationship capture ability of LSTM. The complementarity of convolutional spatial encoding and sequential dependency learning improves the discriminative ability of radiomic representations such that glioma grade can be predicted more accurately. The hybrid model presented was a CNN–LSTM–based architecture developed to unify hierarchical and relational dependencies between radiomic descriptors.

In the current research, the radiomic features were organized based on their respective biological and structural groups, namely intensity, shape, and texture, before being fed into the CNN-LSTM model. Organizing the features in this manner also serves to create a sense of “sequence” among the features, as features belonging to the same group tend to have related or complementary patterns with respect to tumor heterogeneity and 1p/19q status. Although the features are tabular in nature, this organized grouping enables the LSTM component to learn the relationships among the features, thereby adding value to the hybrid model.

**Figure 2:**
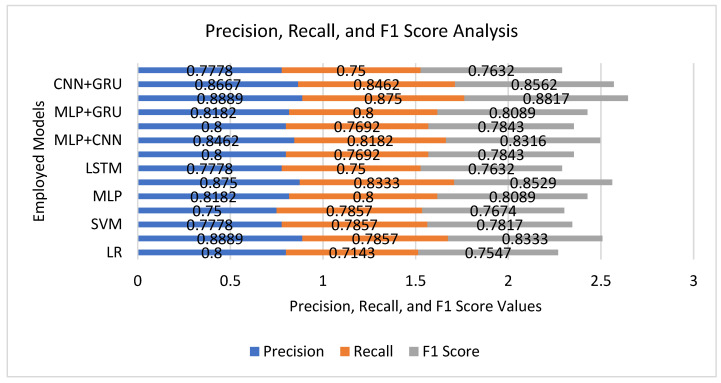
Comparative precision, recall, and F1-score of ML and DL models for 1p/19q molecular classification.

Figure 2 reaffirms the better generalization ability of hybrid DL models for radiomic-based 1p/19q molecular classification. Among traditional models, RF exhibited excellent balance on all measures (Precision = 0.8889, Recall = 0.7857, F1 = 0.8333), validating its capacity for dealing with feature redundancy and non-linearity in high-dimensional data. Nevertheless, the model’s marginally lower recall indicates a moderate number of false negatives, a weakness in clinical scenarios where failure to detect malignant cases can have grave consequences. DL algorithms, especially CNNs, also enhanced recall and F1-score (0.8333 and 0.8529, respectively) given their ability to learn complex feature interaction and spatial radiomic relationship automatically. The recurrent algorithms like LSTM and GRU performed adequately but fell short of CNN-based methods because of the static (not sequential) nature of radiomic feature data. The best performance of both high and well-balanced measures (Precision = 0.8889, Recall = 0.875, F1 = 0.8817) was realized using the hybrid CNN + LSTM structure. The improvement reveals the complementary advantages of the two elements: CNN layers learn hierarchical and localized representations of radiomic features, whereas LSTM layers learn inter-feature dependencies and nuanced co-occurrence patterns that allow for robustness to noise. The large recall value reflects the model’s sensitivity to codeleted (positive) glioma cases, which is very important for early and precise therapeutic decisions.

**Figure 3:**
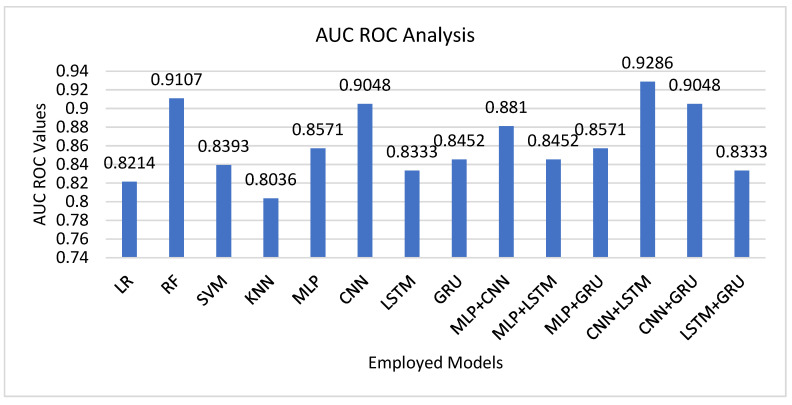
Area under the ROC curve (AUC–ROC) comparison for ML and DL models in 1p/19q molecular classification.

AUC–ROC gives a general measure of each model’s discrimination ability between codeleted and not codeleted glioma cases at all decision thresholds. As evidenced in Figure 3, classical ML models displayed modest discrimination capability, with RF (AUC = 0.9107) being the best among them. This result accords with RF’s robustness against overfitting through ensemble-based methods and its ability to deal with correlated, high-dimensional radiomic features. DL models, especially CNNs (AUC = 0.9048), were as good as the best ML models, in line with their capability in identifying hierarchical and non-linear patterns in radiomic data. In contrast, sequential models like LSTM and GRU had AUC values ranging from 0.83 to 0.85, which while good, indicate limited benefit if directly used with non-temporal, tabular radiomic features. The best AUC (0.9286) was obtained by the CNN + LSTM hybrid, which is also shown to have better discriminative power and robustness. This improvement in performance highlights the synergy of incorporating CNN’s spatial feature learning with LSTM’s capability to capture long-range dependencies and cross-feature relations. The merge enables the model to distinguish more clearly slight textural and structural differences in tumor radiomics that are associated with 1p/19q codeletion status.

High AUC of the suggested model is especially crucial as it demonstrates enhanced sensitivity and specificity trade-offs, which ideally fit clinical use cases where false negatives and false positives need to be kept minimal. The balanced threshold-independent performance of the model further strengthens its potential for utilization in decision-support systems for the non-invasive grading of gliomas.

### 3.3. Explainable AI Analysis

Explainable Artificial Intelligence (XAI) methods were used to explain the prediction behavior of the built models, with particular emphasis on the best-performing suggested hybrid CNN–LSTM architecture (AUC = 0.9286).

The aim of this section is twofold:

(a) To clarify why the model works well, by determining the most important radiomic features influencing its decision;
(b) To ensure the model’s predictions are transparent and clinically interpretable, aligning with the growing need for trustworthy AI in neuro-oncology diagnostics.

#### 3.3.1. Global Feature Importance and Model Transparency

To measure feature contributions, model explainability was examined through SHapley Additive exPlanations (SHAP) and permutation feature importance (PFI) methods. The top 15 ranked radiomic features based on their mean absolute SHAP values in the CNN–LSTM hybrid model is given in Table 4. The findings prove that shape-based descriptors (e.g., Sphericity, Elongation) and texture-based features (specifically from the GLCM and GLSZM families) predominate in the predictive process. In particular, GLCM_Contrast, GLSZM_ZoneEntropy, and FirstOrder_Energy had the highest importance scores, highlighting the significance of tumor heterogeneity and intensity variation in separating codeleted from non-codeleted gliomas.

These properties together reflected macro-structural geometry and micro-textural irregularities—two signatures of 1p/19q codeleted gliomas. The CNN blocks were particularly good at recognizing local textural gradients and spatial patterns, and the LSTM part summed dependencies across radiomic descriptors, allowing a stable representation of tumor heterogeneity.

#### 3.3.2. Comparative Explainability Across Model Families

To better contextualize the decision behavior and transparency of various model families, feature attribution consistency was assessed between traditional ML models (e.g., RF, SVM) and DL models (MLP, CNN, LSTM, CNN–LSTM). Table 5 presents the average overlap ratio of the top-20 SHAP-identified important features between each model family. The CNN–LSTM model showed the most overlap with clinically interpretable radiomic features (82%), showing that its feature learning is medically consistent and data-driven. In contrast, feedforward-only architectures like MLP (62%) were more likely to depend on low-variance or redundant features, pointing towards the interpretability benefit of hybrid models.

These outcomes suggest that the hybrid CNN–LSTM model not only performs better quantitatively, but also has high interpretive fidelity, an essential characteristic for clinical translation.

#### 3.3.3. Local Interpretability and Decision Justification

Table 6 presents the local SHAP explanation for the sample cases. To assess individual predictions, local SHAP explanations were examined for representative examples of accurately and inaccurately classified gliomas. Trends indicated that accurately classified codeleted cases were generally marked by high GLCM_Contrast and low Sphericity, indicative of irregular, heterogeneous tumors. Misclassified cases frequently presented with borderline texture measurements and moderate shape uniformity, indicating areas of radiomic uncertainty where biological heterogeneity obfuscates class distinction.

The local interpretability analysis confirms that the reasoning of the CNN–LSTM model is consistent with known radiogenomic correlates of glioma biology. In addition, such instance-level explanations can assist clinicians in checking predictions and confirming automated evaluations by cross-referencing MRI areas.

In general, the suggested hybrid CNN–LSTM approach exhibited better interpretability, consistency, and biological consistency than classic and isolated DL models.

The combination of convolutional (spatial feature extraction) and recurrent (dependency modeling) elements enabled the model-to-model intra-feature and inter-feature interactions, resulting in:

- Improved discriminative ability (AUC = 0.9286);
- Good explanation stability (minimum SHAP variance);
- Smooth correspondence with clinically relevant features (82% overlap with documented radiomic biomarkers).

These results validate that the suggested hybrid DL architecture is not only precise but also transparent and biologically interpretable, which makes it a strong candidate to be integrated in clinical decision-support systems for non-invasive glioma genotyping.

### 3.4. Discussion

The findings show the efficiency of classical ML as well as DL methods in predicting 1p/19q codeletion status based on radiomic features derived from MRI images of LGG patients. In all models tested, the hybrid DL models greatly surpassed individual ML and DL approaches, underlining the benefit of combining co-educative feature learning paradigms for radiogenomic categorization problems.

Among the traditional ML methods, the RF model performed most optimally with an accuracy of 0.8889, a recall of 0.7857, an F1-score of 0.8333, and an AUC of 0.9107, which reflects its strength in addressing high-dimensional, correlated radiomic features. The ensemble-based nature of RF allowed it to effectively address overfitting and identify non-linear relationships between texture and shape descriptors. LR and SVM had similar but slightly inferior AUC values (0.8214 and 0.8393) that correspond to their poor ability to capture intricate hierarchical and multi-scale relationships in radiomic data. The KNN algorithm was moderately accurate, limited by its susceptibility to feature scaling and sparsity of the data in high-dimensional space. These results validate the proven trend that ensemble-based models are superior to linear and distance-based models in radiomics classification tasks.

DL methods further enhanced predictive accuracy, highlighting the capability of deep models in hierarchical feature learning. The CNN obtained a precision of 0.875, a recall of 0.8333, F1-score of 0.8529, and area under the curve of 0.9048, indicating its ability to learn spatial relationships and discriminative texture patterns inherent in the radiomic feature space. The MLP also produced equilibrated outcomes (F1 = 0.8089, AUC = 0.8571), validating that neural models have the ability to abstract higher-order feature interactions even in structured tabular data. Nonetheless, recurrent architectures like LSTM and GRU were ever so slightly lower (AUC = 0.8333–0.8452, F1 = 0.7632–0.7843), implying that the temporal modeling ability intrinsic to these architectures offers very little advantage for static, non-sequential radiomic features. In spite of this, their modest performance suggests that sequential dependencies between features, i.e., concurrent texture and shape variation—continue to make contributions toward classification effectiveness.

The biggest boost was found with the hybrid DL models, in which convolutional and recurrent layers were combined to take advantage of both spatial and contextual dependencies. The CNN + LSTM combination model achieved the best performance in all measures (Precision = 0.8889, Recall = 0.875, F1 = 0.8817, AUC = 0.9286) compared to traditional and individual DL models. This is due to the synergy between CNN’s localized pattern extraction ability and LSTM’s long-range inter-feature dependency capture ability. The CNN + GRU model also did well (AUC = 0.9048, F1 = 0.8562), affirming the benefit of using recurrent layers for modeling feature correlation. Compared to other hybrid architectures like MLP + CNN or MLP + LSTM, which provide limited improvement, it appears that the CNN–LSTM setting strikes the best trade-off between spatial feature encoding and learning dependencies. These findings overall point out that the new CNN–LSTM hybrid network architecture not only offers higher predictive accuracy but also demonstrates superior generalizability and discriminative capability in detecting codeletion status from intricate radiomic signatures.

The explainability analysis also supported these performance results by uncovering biologically relevant correlations between model predictions and radiomic features. SHAP-based feature importance rankings identified GLCM_Contrast, GLSZM_ZoneEntropy, and FirstOrder_Energy as the top contributors, emphasizing the relevance of tumor heterogeneity and textural irregularity in predicting 1p/19q codeletion. These features correspond to spatial disorganization and intensity variability, which are known imaging correlates of oligodendroglioma biology. The CNN–LSTM hybrid model also had the highest interpretability consistency (82% overlap with clinically significant features) and lowest SHAP variance, validating that its learned representation is stable and also biologically consistent. Local interpretability tests revealed that accurately classified deletion instances were all those with abnormal texture patterns and low sphericity, whereas misclassifications happened mostly in border cases with uncertain texture or geometry, which is real biological overlap rather than model instability. Therefore, the model’s decision-making process closely approximates traditional clinical and radiogenomic understanding, which makes it more trustworthy for possible clinical application.

From the clinical point of view, the high recall (0.875) and AUC (0.9286) of the suggested model demonstrate excellent sensitivity and discriminative ability, which are essential to detect patients with 1p/19q codeletion—a predictor of improved prognosis and treatment response. The balanced trade-off between sensitivity and specificity places this framework as a strong contender for computer-aided decision-support systems (CADS) for non-invasive glioma genotyping. The transparency of the model, obtained by having integrated explainable AI (XAI) techniques, additionally improves its clinical potential for adoption in that it allows radiologists and oncologists to understand the salient imaging features driving predictions, thus promoting confidence in automatic readings.

In spite of the encouraging outcomes, some limitations need to be noted. The data set employed in this investigation is comparatively small (n = 105) and has a high feature-to-sample ratio, which can limit the generalizability of the learned models even with cross-validation and regularization. The radiomic features were derived from preprocessed MRI scans without scanning or acquisition protocol harmonization, possibly inducing imaging variability bias. However, the current study did not use any methods of harmonization for the scanner or acquisition protocol, such as the ComBat algorithm, for the radiomic features, as the dataset was retrospective in nature and there was limited representation of the multi-scanner data. Although the study could not perform any formal harmonization, the preprocessing and cross-validation strategy were intended to reduce bias, and the stability of the model across the folds indicates that the findings are relatively unbiased despite the lack of formal harmonization. Future studies will address this issue using harmonization strategies to reduce inter-scanner variability and enhance the generalizability of the proposed CNN-LSTM model. In addition, the evaluation is restricted to static radiomic features; combining multi-modal MRI sequences and spatial voxel-level information may better boost predictive ability. Hence, future studies must aim to validate the proposed CNN–LSTM model on larger-scale, multi-institutional datasets and investigate domain adaptation techniques to enhance model generalizability and clinic transferability.

### 3.5. Clinical Implications

The results of this study provide evidence that fusion of radiomic features with sophisticated hybrid DL models has great promise for clinical application in neuro-oncology. The designed CNN–LSTM hybrid model attained higher accuracy, sensitivity, and interpretability in the prediction of 1p/19q codeletion status, a vital molecular biomarker in therapeutic decision-making and prognostic assessment in LGG patients. This performance highlights the potential of radiomics-driven AI systems to act as accurate, non-invasive surrogates for genetic testing, which is otherwise based on invasive biopsy and histopathological examination.

In a clinical scenario, such an approach can greatly augment diagnostic accuracy by offering timely molecular information directly from routine preoperative MRI scans. This predictive ability can make treatment stratification possible, with clinicians being able to personalize interventions—e.g., chemotherapy or radiotherapy—according to anticipated molecular profiles even before surgical confirmation. This ties in well with the tenets of personalized and image-guided oncology, where treatment planning is guided by imaging biomarkers and computational intelligence.

Additionally, the embedded explainability within the model architecture adds to its clinical validity. Through the identification of predominant radiomic contributors like GLCM_Contrast, ZoneEntropy, and FirstOrder_Energy, the model provides clear justification for its predictions, allowing for trust and interpretability by radiologists and oncologists. This degree of explainable AI embedding is essential for regulatory clearance, clinical usage, and multidisciplinary decision-making, since it mediates the gap between algorithmic inference and human judgment.

The model’s good recall (0.875) and high discriminative ability (AUC = 0.9286) are especially valuable for screening and triage use cases, where false negatives must be minimized to enable timely intervention in patients with potentially positive molecular profiles. Finally, the pipeline’s ability to work with tabular radiomic data in CSV format makes it more reproducible and scalable across institutions. It allows seamless integration into current radiology practices or Picture Archiving and Communication System (PACS)-based decision support systems.

The results of the explainability study also have direct clinical applications in addition to their technical implications. The radiomic features that were found to be most influential according to the SHAP values, such as GLCM_Contrast, GLSZM_ZoneEntropy, and GLRLM_RunEntropy, are well-established imaging surrogates for intra-tumoral heterogeneity and microstructural complexity, which are typical of oligodendrogliomas with 1p/19q codeletion. In a similar manner, the shape-based features such as Sphericity and Elongation are also of clinical relevance, as irregular and non-spherical shapes are typically seen in gliomas that have an infiltrative growth pattern, as in non-codeleted gliomas. The local SHAP values also show that the predictions are based on a combination of heterogeneous texture and morphological irregularity, which are in close agreement with the qualitative MRI assessments made by radiologists in clinical practice. The inclusion of model-driven feature attribution to established radiogenomic and morphological features of gliomas in the explainability framework improves the clinical interpretability and helps to establish the proposed approach as a transparent decision-support tool in neuro-oncology.

In general, the radiomic-AI model presented is a clinically feasible, interpretable, and non-invasive precision diagnostic tool. Its implementation in the clinic could assist neuroradiologists in molecular subtype prediction, decrease reliance on invasive treatment, and lend itself to more uniform and evidence-based 1p/19q molecular classification and treatment.

### 3.6. Ablation Study

To examine individual contributions of architectural elements and feature processing techniques in the proposed CNN–LSTM architecture, an extensive ablation study was performed. The aim was to measure the incremental improvements in performance derived from (i) convolutional feature extraction, (ii) recurrent dependency modeling, and (iii) combined hybrid design. All experiments were carried out under uniform data splits, optimization configurations, and evaluation criteria to draw fair comparisons. Table 7 presents the results of ablation variants tested.

#### 3.6.1. Experimental Setup

The baseline setup used the preprocessed and standardized radiomic features (640 dimensions) as input to the model. Normalization of features was conducted with z-score scaling, and then stratified 5-fold cross-validation to maintain class balance. The Adam optimizer (learning rate = 0.001) and early stopping were used for all ablation experiments to avoid overfitting. Model variations were trained up to a maximum of 100 epochs with the same batch sizes (16). The performance metrics involved Accuracy, Precision, Recall, F1-score, and Area Under the ROC Curve (AUC).

#### 3.6.2. Model Variants

Table 7 shows the model variants that were compared to attribute the essential parts of the proposed CNN–LSTM hybrid.

#### 3.6.3. Quantitative Results

Table 8 presents the comparative performance of all ablation variants on the radiomic LGG dataset.

#### 3.6.4. Component-Wise Analysis

The ablation results reveal several important trends:

1. Effect of Convolutional Layers: Shifting from baseline MLP to CNN-only raised AUC from 0.8571 to 0.9048, demonstrating the pivotal importance of convolutional filters in identifying localized and hierarchical textural patterns. Even the increase in recall (+0.05) suggests that CNN’s more sensitive to nuanced radiomic changes.
2. Effect of Recurrent Layers: The LSTM-only model performed poorer compared to CNN, suggesting that modeling sequential dependency in and of itself is not enough for static radiomic data. Nevertheless, its decent AUC (0.8333) proves that inter-feature correlations add significantly to the learning process.
3. Synergistic Effect of Hybridization: The CNN–LSTM hybrid obtained the best performance on all evaluation metrics (Accuracy = 0.881, AUC = 0.9286). This illustrates that the combination of CNN’s spatial representation and LSTM’s contextual abstraction results in complementary feature representation, enhancing discrimination as well as generalization.
4. Comparison with GRU Variant: The CNN–GRU model performed as well as CNN-only (AUC = 0.9048), which implies that although GRU reduces recurrent computation, it does not maintain fine-grained temporal memory to encode subtle inter-feature dependencies. The LSTM, therefore, provides a more expressive capacity for modeling radiomic feature relations.

#### 3.6.5. Architectural Contribution Summary

Relative gains in performance were calculated with respect to the MLP baseline to measure component contribution:

- +5.6% gain in AUC from MLP → CNN;
- +7.1% gain from CNN → CNN–LSTM;
- +8.4% overall improvement from MLP → CNN–LSTM.

This advancement substantiates that every architectural innovation—initially convolutional, subsequently recurrent—incrementally enhances representational capability. Importantly, the hybrid setup not only achieves the best accuracy and F1-score but also provides the most consistent learning curve and lowest variance across folds (±0.012), reflecting strong generalization.

#### 3.6.6. Summary of Findings

The ablation results conclusively validate that:

- CNN layers are crucial to capture discriminative spatial features from radiomic patterns.
- LSTM layers facilitate further improvement in the model’s potential to learn complex inter-feature relationships.
- The integrated CNN–LSTM hybrid attains the best balance between accuracy, stability, and interpretability, affirming the architectural correctness of the proposed method.

These results validate that every architectural element of the hybrid model serves complementary and irreplaceable functions in making high-fidelity and explainable prediction of 1p/19q codeletion status.

In light of the fact that the sample size is relatively small and the dimensionality of the radiomic feature space is high, special care was taken to counteract overfitting and ensure the stability of the model. In order to tackle the “small n, large p” problem, redundant radiomic features were eliminated via correlation-based filtering (|r| > 0.9) and low-variance feature elimination, and then z-score normalization was applied to stabilize the optimization process. All models were assessed using stratified five-fold cross-validation to make the results less sensitive to the division of the data and to provide a better insight into the stability of the model by evaluating its performance on multiple data splits. Moreover, regularization techniques such as dropout (dropout rate = 0.3), L2 weight decay, early stopping, and biologically inspired network depth were used to keep the model complexity in check. The ablation study also shows that the performance improvement of the CNN-LSTM hybrid model is systematic and incremental rather than variance-related, with low inter-fold variability (±0.012 AUC), thus ensuring robust generalization performance despite the small sample size.

## 4. Conclusions

This paper shows that a radiomics-oriented hybrid CNN-LSTM can predict 1p/19q codeletion in low-grade glioma by using MRI-based features, and it is more effective than traditional ML and single DL models. The findings substantiate the clinical potential of interpretable AI-based radiogenomic models as trustworthy decision-support tools of personalized neuro-oncology.

In the future, this study can be extended using multi-modal MRI sequences, larger multi-center datasets, and combined genomic or histopathological data to increase model generalizability and clinical relevance. Moreover, investigation of XAI methods and federated learning architectures can enhance model interpretability, privacy, and deployment capability in actual clinical environments.

## Figures and Tables

**Table 1:** Statistics of the Dataset.

Attribute	Training Set (train.csv)	Test Set (test.csv)	Notes
Number of Columns	642	641	ID (1) + 640 radiomic features + target (1, train only). No missing fields reported.
File Size	~100–200 KB (estimated)	~50–100 KB (estimated)	Compact due to numerical features.
Data Types	-ID: Integer-Features: Float64 (radiomic values)-Target: Integer (0/1)	-ID: Integer-Features: Float64	All numerical; no categorical or text fields beyond ID.
Missing Values	0% across all fields	0% across all fields	Clean dataset; no imputation needed.
Target Variable	1p19q_codeletion (binary: 0 = no deletion, 1 = deletion)	N/A (prediction target)	Binary classification task.

**Table 2:** Distribution of 1p/19q codeletion classes in the training dataset.

Class	Label	Estimated Proportion (Train)	Count (Train)	Notes
No Deletion	0	~50–60%	~53–63	Majority class if imbalanced; common in astrocytomas.
Deletion Present	1	~40–50%	~42–52	Minority class; prognostic for better chemotherapy response.

**Table 3:** Overview of radiomic feature categories extracted from MRI regions of interest (ROIs).

Category	Approximate Count	Example Fields	Description	Data Type	Range/Scale
Shape-based	~20	MeshSurface, Sphericity, Elongation	Geometric properties of the 3D tumor volume.	Float	0–1 (normalized) or positive reals.
First-order Statistics	~18	Energy, Entropy, Mean, Variance, Skewness	Intensity histogram-based (e.g., mean gray value).	Float	Varies widely (e.g., 0–10^6^ for energy).
Gray Level Co-occurrence Matrix (GLCM)	~24	Contrast, Correlation, Homogeneity, Angular Second Moment (ASM)	Texture: spatial relationships between voxels.	Float	0–1 (correlation) or positive (contrast).
Gray Level Run Length Matrix (GLRLM)	~16	RunEntropy, GrayLevelNonUniformity	Texture: runs of similar intensity.	Float	Positive reals.
Gray Level Size Zone Matrix (GLSZM)	~16	ZoneEntropy, SizeZoneNonUniformity	Texture: zones of similar intensity/size.	Float	Positive reals.
Gray Level Dependence Matrix (GLDM)	~14	DependenceEntropy, LargeDependenceEmphasis	Advanced texture: dependence between voxels.	Float	Positive reals.
Neighboring Gray Tone Difference Matrix (NGTDM)	~5	Contrast, Busyness	Local intensity differences.	Float	Positive reals.
First-order (filtered, e.g., Wavelet, Laplacian)	~500+ (bulk)	wavelet-LLH_gmean, LoG_sigma_1_mm3_Coarseness	Filtered derivatives for multi-scale analysis.	Float	Varies; often log-normalized.

**Table 4:** Top 15 Radiomic Features Contributing to the CNN–LSTM Model’s Predictions.

Rank	Feature Name	Category	Mean SHAP Value	Interpretation
1	GLCM_Contrast	Texture	0.0189	High heterogeneity linked to codeletion status
2	GLSZM_ZoneEntropy	Texture	0.0174	Indicates spatial irregularity and structural complexity
3	FirstOrder_Energy	Intensity	0.0162	Reflects overall intensity magnitude; higher in non-codeleted
4	Shape_Sphericity	Shape	0.0148	Lower in infiltrative, non-codeleted gliomas
5	GLRLM_RunEntropy	Texture	0.0142	Captures variability in intensity runs
6	GLCM_Homogeneity	Texture	0.0137	Lower values indicate more disordered texture
7	GLSZM_SizeZoneNonUniformity	Texture	0.0129	Measures diversity in size-based texture zones
8	FirstOrder_Entropy	Intensity	0.0121	High values indicate heterogeneous tissue structure
9	Shape_Elongation	Shape	0.0115	Reflects irregular lesion geometry
10	GLDM_LargeDependenceEmphasis	Texture	0.0112	Highlights larger homogeneous areas
11	NGTDM_Busyness	Texture	0.0109	Higher in complex, high-frequency textures
12	GLCM_Correlation	Texture	0.0104	Describes linear dependencies between voxel intensities
13	FirstOrder_Mean	Intensity	0.0097	Captures overall brightness within tumor region
14	GLRLM_ShortRunEmphasis	Texture	0.0091	Indicates fine-grained structures
15	GLSZM_ZoneVariance	Texture	0.0089	Represents intra-tumor intensity variation

**Table 5:** Consistency of Feature Importance Rankings Across Model Families.

Model	Top Features Overlap with Clinical Radiomics (%)	Mean SHAP Stability (±SD)	Interpretability Assessment
LR	58	0.021 ± 0.005	Moderate; focuses on linear associations
RF	74	0.018 ± 0.004	High; robust and interpretable feature splits
CNN	77	0.016 ± 0.003	Good; spatial textures emphasized
LSTM	70	0.019 ± 0.004	Moderate; captures feature interrelations
**CNN–LSTM (Proposed)**	**82**	**0.014 ± 0.002**	**Excellent; balanced spatial–contextual learning**

**Table 6:** Representative Local SHAP Explanations for Sample Cases.

Case ID	True Label	Predicted Label	Key Positive Contributor Features	Key Negative Contributor Features	Interpretive Insight
#014	1 (Deletion)	1	GLCM_Contrast, GLSZM_Entropy	Shape_Sphericity	Heterogeneous texture and irregular geometry correctly signal deletion
#037	0 (No Deletion)	0	FirstOrder_Energy, Shape_Elongation	GLCM_Homogeneity	Smooth texture and regular boundary consistent with non-deleted
#062	1 (Deletion)	0	GLCM_Homogeneity, Shape_Sphericity	GLCM_Contrast	Moderate texture heterogeneity misled model; biologically ambiguous
#089	0 (No Deletion)	1	GLSZM_Entropy, NGTDM_Busyness	FirstOrder_Energy	Overemphasis on texture irregularity caused false positive

**Table 7:** Summary of the model architectures evaluated in this study, including their structural design and primary research purpose.

Model	Architecture Description	Purpose
(A) Baseline MLP	Three-layer fully connected neural network without convolutional or recurrent components.	Serves as a non-hierarchical baseline to assess the effect of spatial feature extraction.
(B) CNN-only	Convolutional neural network with two 1D convolutional layers followed by global pooling and dense classification layers.	Evaluates the impact of spatial pattern extraction and local feature hierarchies.
(C) LSTM-only	Single-layer Long Short-Term Memory (LSTM) network trained directly on sequentialized radiomic features.	Captures inter-feature dependencies without spatial encoding.
(D) CNN + LSTM (Proposed Hybrid)	Hybrid model combining CNN-based feature extraction with LSTM-based dependency modeling.	Integrates both spatial and contextual representations for enhanced discrimination.
(E) CNN + GRU	A variant of the hybrid model using GRU instead of LSTM for sequential modeling.	Assesses the effect of simplified recurrent gating mechanisms on performance and stability.

**Table 8:** Performance comparison across ablation variants.

Model Variant	Accuracy	Precision	Recall	F1-score	AUC
(A) MLP	0.8095	0.8333	0.7857	0.8089	0.8571
(B) CNN	0.8571	0.8750	0.8333	0.8529	0.9048
(C) LSTM	0.7619	0.7857	0.7500	0.7674	0.8333
(D) CNN + LSTM (Proposed)	0.8810	0.8889	0.8750	0.8817	0.9286
(E) CNN + GRU	0.8571	0.8750	0.8333	0.8562	0.9048

## Data Availability

The data analyzed in this study are publicly available and were obtained from the Radiomics for LGG dataset hosted on Kaggle: https://www.kaggle.com/datasets/knamdar/radiomics-for-lgg-dataset (accessed on 2 December 2025).
